# In Situ Measurement of Cyclic Plastic Zone and Internal Strain Response of Q&P Steel near Fatigue Crack Tip Region Based on Micro-DIC

**DOI:** 10.3390/ma15176114

**Published:** 2022-09-02

**Authors:** Hongli Gao, Zhiyuan Lin, Xinwei Huang, Hongbin Shang, Jingsong Zhan

**Affiliations:** Key Laboratory of Special Equipment Manufacturing, Advanced Processing Technology of the Ministry 5 of Education, Zhejiang University of Technology, Hangzhou 310023, China

**Keywords:** in situ measurement, cyclic plastic zone, micro-DIC, image stitching and matching, fatigue crack, strain loop and response

## Abstract

The shape and internal dynamic response characteristics of the plastic zone near the fatigue crack tip region, especially the cyclic plastic zone (CPZ), are the main factors affecting the fatigue crack initiation and propagation behaviors of ductile metal materials. The existing methods for characterizing the CPZ have some problems, which include the complexity of the process, the difficulty of achieving in situ measurement, and the inability to characterize the dynamic response in the CPZ during the crack propagation process. Therefore, a novel method is proposed for the in situ measurement of the CPZ near the crack tip region based on image stitching and matching algorithms, a load–strain loop curve characteristic judgement algorithm, and the microscopic digital image correlation (DIC) method. A microscopic camera and a macroscopic camera are used to simultaneously capture the micro crack tip speckle images and the global crack image of the two sides of the Compact Tension (CT) specimen for calculating in situ crack length and crack tip strain fields. The proposed method was performed and verified by a fatigue crack growth (FCG) test and micro-hardness experiments with Quenching and Partitioning 980 (Q&P980) steel, and the results show that the method is feasible because the maximum error is less than 5%. A “butterfly wings” shape of the CPZ and a strain concentration phenomenon in the CPZ of the Q&P980 were observed. Moreover, as the fatigue crack propagates, the area of the CPZ and the degree of the strain concentration increase gradually. This method, which can obtain the in situ and tracking measurements of the crack tip CPZ, will help to increase our understanding of CPZ characteristics, the FCG mechanism, and the behavior of Q&P steel and the plastic metal materials similar to Q&P steel.

## 1. Introduction

The plastic zone refers to the area where the material is plastically deformed at the tip of a growing crack subject to cycle load [1]. Under the action of alternating load, the plastic deformation occurs near the crack tip region and forms a tiny plastic zone in ductile metal materials, such as Q&P steel, and there is a smaller cyclic plastic zone embedded in the plastic zone [2]. As we all know, the fatigue cracks of ductile materials cannot propagate across virgin material; the propagation always occurs inside material that has been previously damaged by moving cyclic plastic zones, where the fatigue damages are most severe and which are related to the local strain ranges. As the main failure mode of metal mechanical parts, fatigue failure will occur when fatigue cracks continuously propagate. Some existing studies show that fracture behavior is closely related to the initiation and propagation of a fatigue crack in materials [3,4,5]. According to previous research, the shape, internal deformation, and mechanical response characteristics of the cyclic plastic zone are the main factors affecting fatigue crack initiation and propagation behaviors and can also be used to reveal the microscopic mechanism of the material fatigue failure process [6,7,8].

Therefore, the study of the plastic zone, especially the cyclic plastic zone, has become a research hotspot in recent years, in which the size and shape measurement is the key. In 1997, Agah et al. [9] made some summaries on the measurement methods of the plastic zone. However, most existing studies on the plastic zone are about the monotonic plastic zone (PZ) rather than the CPZ. This is because the size of the CPZ is much smaller than that of the PZ and is very difficult to separate from the plastic zone [10], which also leads to greater difficulty in obtaining an accurate CPZ measurement. The methods that are used to characterize the PZ or the CPZ can be divided into two categories: one is theoretical analysis combined with the numerical simulation, and the other is actual measurement and observation.

For the PZ, some studies try to characterize the size and shape of the PZ by means of theoretical analysis, numerical simulation, and 2D or 3D finite element analysis (FEA) so as to further analyze the fatigue mechanism of the material [11,12,13,14,15,16]. However, these methods have many limitations, especially in terms of actual observation. The methods of measuring the plastic zone for actual observation include the strain gauge method, etching method, hardness method, etc. [17,18,19,20]. However, the above methods can only characterize the PZ under contact and ex situ conditions. Fortunately, with the emergence and development of global non-contact DIC/micro-DIC technology, which has higher measurement accuracy and practicability, there are more and more works to study the plastic zone by combining the DIC/micro-DIC and theoretical models [21,22,23,24], so non-contact in situ PZ measurement can be achieved. However, it is still difficult to measure the CPZ and internal dynamic response in situ.

For the CPZ, Park et al. [25] attempted to graphically verify the effect of CPZ size on the fatigue crack growth rate and analyzed the factors affecting the size of the CPZ using the FEA method. Chikh et al. [26] studied the evolution of the fatigue crack grown rate (FCGR) of 12NC6 steel and the effect of the CPZ on this evolution using the FEA method. Bathias and Pelloux [27] determined the dimensions of a monotonic plastic zone and the CPZ of austenitic stainless steel by the micro-hardness method. Gonzáles et al. [28] used a stereo microscope coupled with a 3D DIC system and combined with the 3D FEA method to measure strain ranges inside the CPZ ahead of a fatigue crack tip. Based on the current research, it can be seen that there is almost no accurate method used for in situ CPZ measurement, especially to meet the requirement of dynamic tracking of the fatigue crack tip position to measure the CPZ when the crack length is beyond the field of view of the microscopic camera.

In this paper, a novel method for the in-situ measurement of the CPZ near the crack tip based on micro-DIC is proposed in order to fill the gap in the field of in situ CPZ measurement in ductile metal materials similar to Q&P steel during the FCG process. This method combines micro-DIC with the reference image stitching and target image matching algorithms, as well as the load–strain loop curve characteristic judgment algorithm to measure the fatigue crack tip CPZ at any length in the stable crack propagation stage. The reference image stitching and target image matching algorithms, which were introduced in our previous work [29] in detail, are mainly used to solve the measurement problem, that is, that the micro-camera cannot be directly used to measure the micro crack tip strain fields of the long crack. In our previous work [29], we mainly obtained in situ online strain fields data near the crack tip region of short and long cracks. The obtained strain fields data are an important basis for the current research carried out in this paper.

The main research contributions of this paper are as follows:

(1) The load–strain loop curves of the crack tip region are obtained by coupling the in situ strain field data with the related load data.
(2) The boundary of the CPZ is obtained by the load–strain loop curve characteristic judgement algorithm.
(3) In order to verify the accuracy of the measured results, the FCG test and micro-hardness experiment are performed with Compact Tension (CT) specimens of Q&P980 material, which is representative of the third generation of advanced high-strength steel (AHSS), proposed by Speer et al. in 2003 [30].
(4) The evolution law of the shape and size of the measured CPZ are analyzed, and the strain response characteristics in the CPZ are discussed.

This method overcomes the problems of CPZ in situ measurement and realizes the non-contact and in situ dynamic tracking CPZ measurement. It supplies strong support for deeper research on the fatigue crack growth behavior and the mechanism of Q&P steel and ductile metal materials similar to Q&P steel.

## 2. In Situ Crack Tip CPZ and Internal Strain Response Measurement System

### 2.1. Overall Measurement Method

**Figure 1:**
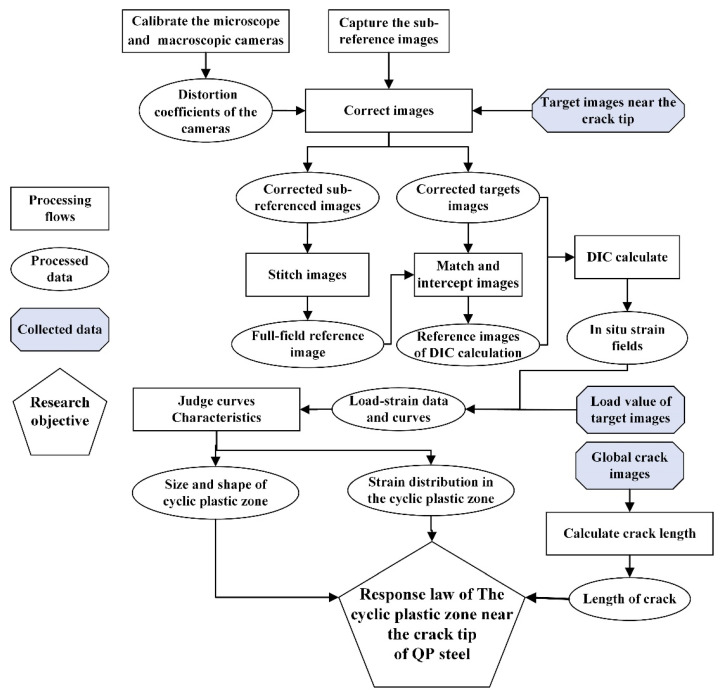
A flow chart of the in situ CPZ and internal response measurement method.

A chart of the measurement method proposed in this paper is shown in Figure 1, which mainly includes six processing stages: calibrating the cameras, capturing reference images, correcting images, stitching images, matching and intercepting images, calculating the crack tip region strain field and crack length, and judging load–strain loop curve characteristics of the crack tip region. The macroscopic and microscopic cameras were calibrated with Zhang’s calibration method [31]. In the process of calibration, the calibration boards were made, and the calibration board images with different angles needed to be captured. The main purpose of Zhang’s calibration method is to calculate the distortion parameters of the camera system by spatial transformation based on the images of calibration plates with different angles. The captured images were corrected using the distortion coefficients in the processing of correcting images. The corrected sub-reference images were stitched to obtain the full-field reference image. A global crack image and target images were collected during the fatigue crack growth test. The corrected target images were used to match with the full-field reference image. The strain fields data at the crack tip were calculated using the micro-DIC technique. The CPZ were distinguished by the load–strain loop curve characteristic judgement algorithm, which judged the load–strain curve of every pixel within the crack tip region strain field, and the crack’s length was obtained by processing the global crack images.

### 2.2. Materials and Specimen

**Figure 2:**
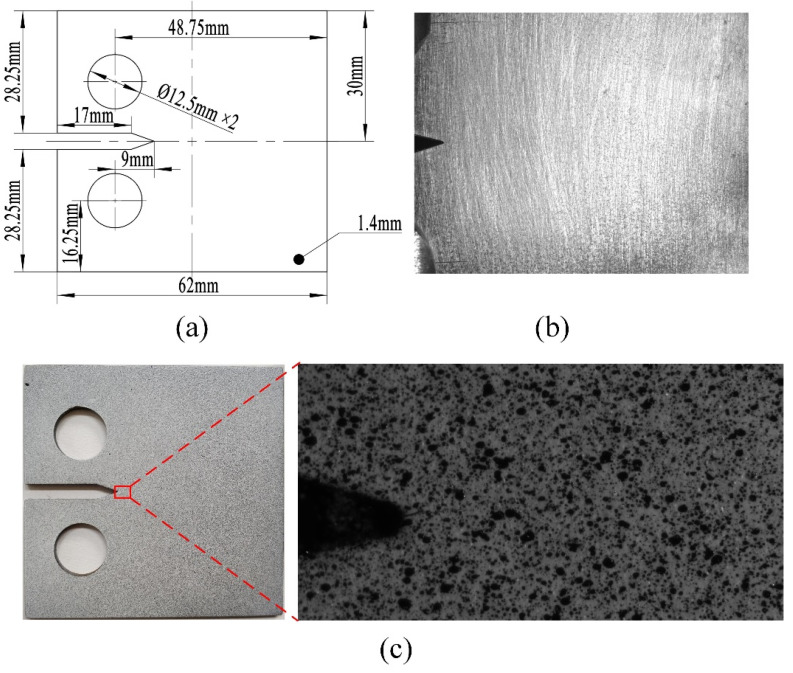
CT specimen information: (**a**) Geometric parameters of the CT specimen; (**b**) global crack image of the CT specimen; (**c**) digital speckle image of the CT specimen.

The specimen used in this research was a CT specimen with a model I crack made of Q&P980 material; the parameters of the specimen’s geometry are shown in Figure 2. Figure 2a shows the dimensions of the CT specimen. One side of the CT specimen is polished to a smooth surface with diffuse effect, as shown in Figure 2b, and the other side is made into a speckle surface with uniform speckles attached, as shown in Figure 2c. The speckle-making tool is a professional spray pen.

In order to obtain the mechanical properties of Q&P980, the uniaxial tensile test was performed, and the test specimen was a dog-bone specimen made of Q&P980 steel. The mechanical properties as shown in Table 1 were obtained by fitting and modifying the stress–strain curve combined with the Ramberg–Osgood (R-O) model [32]; the data processing details were introduced in our previous work [29].

Speckle quality of a CT specimen surface directly affects the result of DIC calculation. The average gray second derivative and average gray gradient are two important indexes for speckle quality evaluation. The calculation formulas are shown in (1) and (2):

(1) $$\delta_{f}=\frac{\sum_{i=1}^{W}\sum_{j=1}^{H}\sqrt{f_{x}{\left(x_{ij}\right)}^{2}+f_{y}{\left(y_{ij}\right)}^{2}}}{W*H}$$

(2) $$\omega_{f}=\frac{\sum_{i=1}^{W}\sum_{j=1}^{H}\sqrt{f_{xx}{\left(x_{ij}\right)}^{2}+f_{yy}{\left(y_{ij}\right)}^{2}}}{W*H}$$

where *H* and *W* are the pixels height and pixels width of the image, respectively.$\;f_{x}{\left(x_{ij}\right)}^{2}$, $f_{y}{\left(y_{ij}\right)}^{2}$ are the grayscale derivatives of pixel $x_{ij}$ in x and y directions,.$\;f_{xx}{\left(x_{ij}\right)}^{2}$, $f_{yy}{\left(y_{ij}\right)}^{2}$ are the grayscale second derivatives of pixel $x_{ij}$ in x and y directions. The larger $\delta_{f}$ is given priority. Under the condition that $\delta_{f}$ is equal, the smaller $\omega_{f}$ is given priority.

### 2.3. In Situ CPZ Measurement System Components and Multi-Scale Images Acquisition

**Figure 3:**
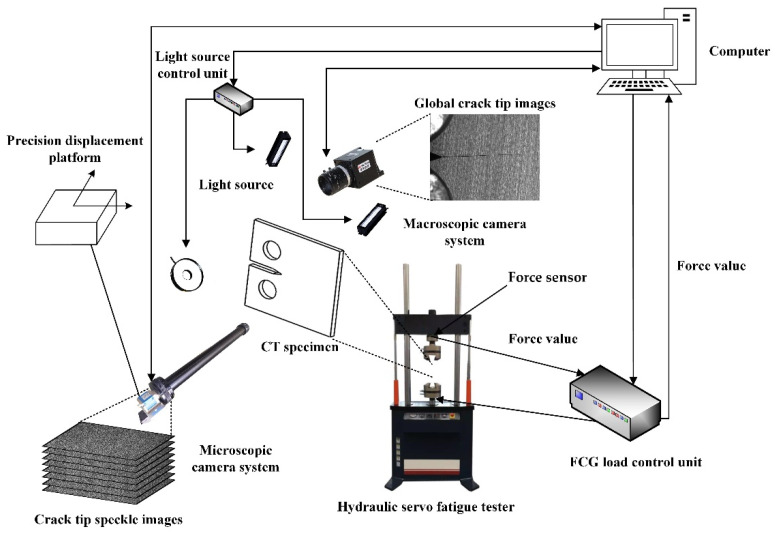
In situ CPZ and internal response measurement system components [29].

**Figure 4:**
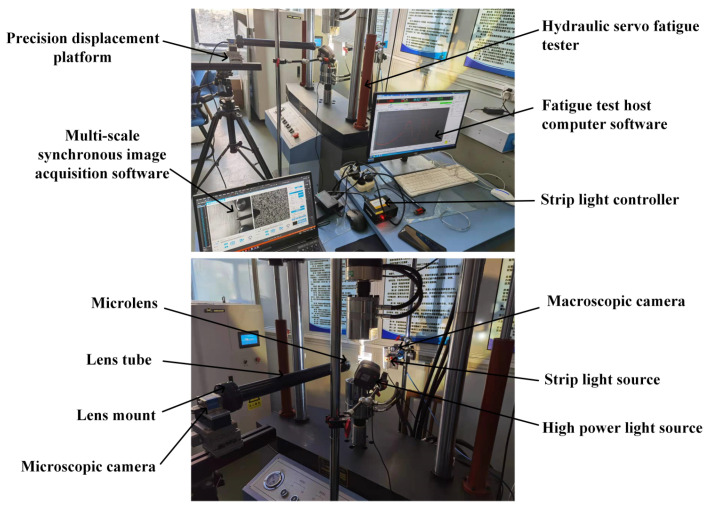
Experimental set-up [29].

As shown in Figure 3 and Figure 4, the hardware components of the in situ measurement system and experimental set-up for CPZ in situ measurement are the same as those in our previous work [29]. However, in addition to image calibration software, multi-scale image synchronous acquisition software, image stitching and matching software, and VIC-2D software, the in situ CPZ measurement system also includes software for measuring the CPZ and internal response at the crack tip.

The parameters of the macro- and micro-cameras used in the proposed measurement system are shown in Table 2.

**Figure 5:**
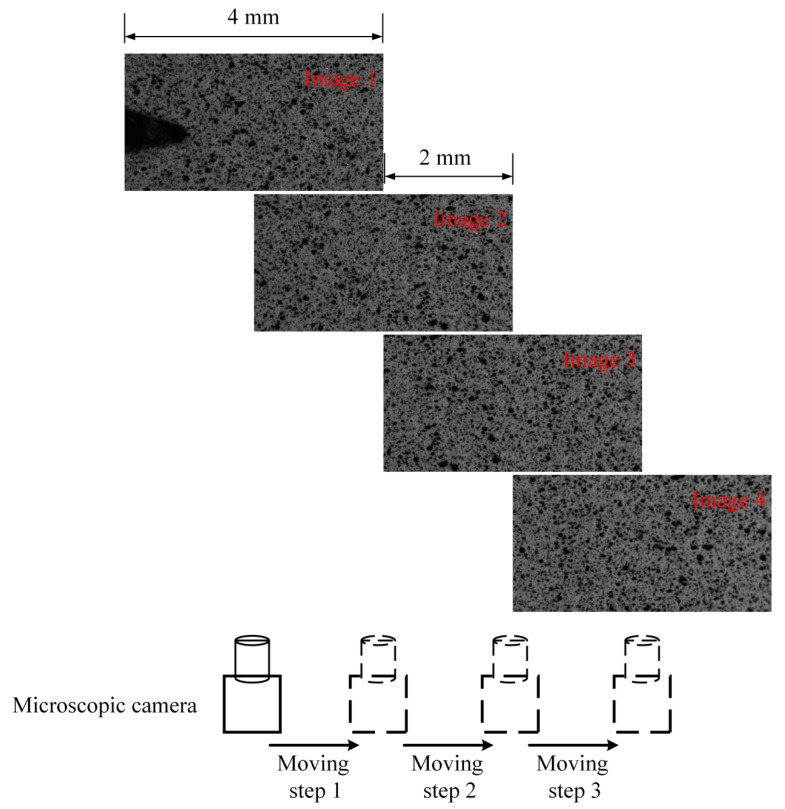
Sub-reference images collection.

**Figure 6:**
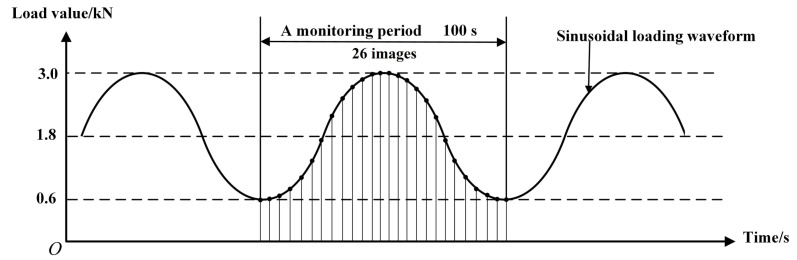
Schematic diagram of acquisition point layout of speckle images in a load cycle.

The multi-scale fatigue crack and speckle images acquisition process of the proposed method was roughly divided into two steps as follows:

(1) Capturing sub-reference images before the FCG test: The microscopic camera moving step was set to be 2 mm, which is half of the horizontal field of view. The fatigue crack growth length was set to be 8 mm. Four sub-reference images, as shown in Figure 5, were collected by moving the microscopic camera shown in Figure 4. After the four speckle images were captured, the position of the microscopic camera needed to be reset to the initial position.
(2) Capturing target images during the FCG test: After setting test parameters, the FCG test was started. The FCG test load parameters are shown in Figure 6, and the fatigue testing frequency was 8 Hz. Under these test conditions, the fatigue crack could grow stably until the fatigue crack length was close to 15 mm according to previous experience. In order to ensure that the collected images could be used to calculate the strain field, when the position of the crack tip was close to 1/3 of the right edge of the microscopic field of view, the position of the microscopic camera needed to be adjusted. Finally, six groups of images with cycle numbers of 10,000, 15,000, 20,000, 22,500, 25,000, and 27,000 were collected. In the process of image acquisition, the test frequency needed to be reduced to 0.01Hz, and 1 macroscopic global crack image and 26 microscopic speckle images were collected in each monitoring period and saved on the computer in a defined order. The arrangement of acquisition points for 26 speckle images are shown in Figure 6.

**Figure 7:**
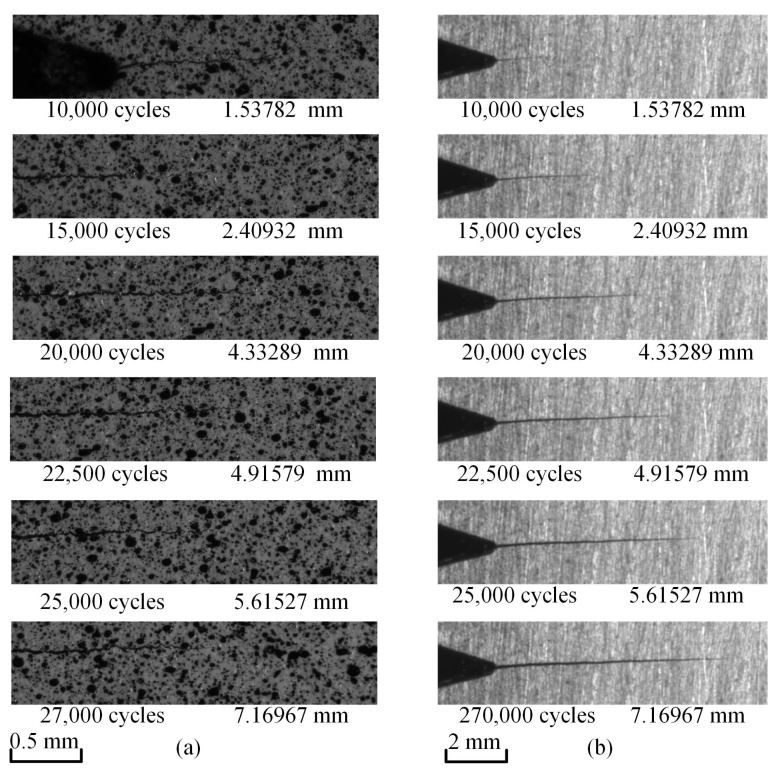
Maximum load point images corresponding to different cycle numbers: (**a**) Speckle images; (**b**) global crack images.

Among the six groups of images collected, the speckle images and the global crack images at the maximum load points are shown in the Figure 7.

## 3. In Situ Measurement Algorithms of CPZ and Internal Response at the Crack Tip

### 3.1. Measurement Algorithms of CPZ and Internal Response near the Crack Tip Based on DIC

**Figure 8:**
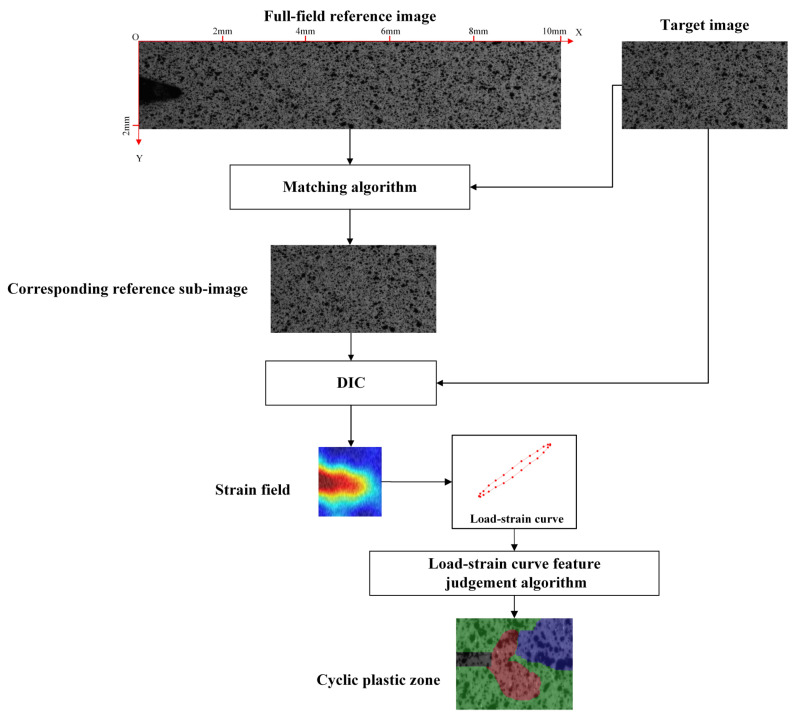
The measurement process of CPZ near the crack tip region.

The measurement flow of CPZ based on DIC is shown in Figure 8, in which the core technologies are DIC and the load–strain curve characteristic judgment algorithm. In order to calculate the in situ strain field at the different crack lengths [29], it was necessary to use the image stitching and matching algorithm to obtain the full-field reference image and the reference images corresponding to the target images, as shown in Figure 8. Our previous work [29] introduced the specific implementation and the accuracy verification details of the sub-reference image stitching and target image matching algorithm. Finally, the target images and reference sub-images were used for DIC calculation to obtain the strain fields of the crack tip region. According to this method, the strain fields data of different crack lengths in the process of crack propagation can be calculated. The load–strain curve of each pixel within the strain field can be obtained by coupling the load value information recorded during the image acquisition. The shape and size of the CPZ were measured by the load–strain curve characteristic judgement algorithm.

**Figure 9:**
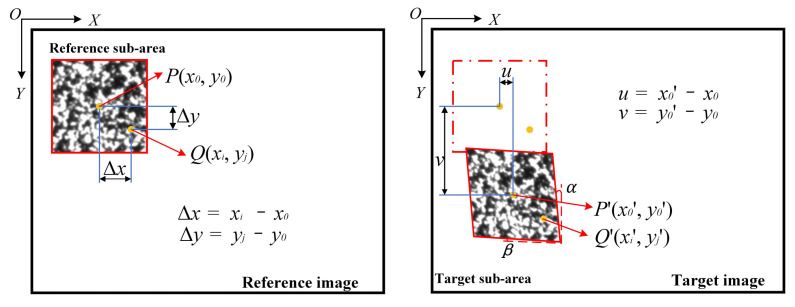
DIC calculation principle.

DIC calculation is the key step in obtaining the load–strain loop curve. Compared with holographic interferometry [33], Moiré interferometry [34,35], and laser speckle photography technology [36,37], DIC has better applicability in non-contact deformation measurement. The principle of the DIC algorithm is shown in Figure 9 [13]. The main process is to calculate the deformation displacement fields of the target sub-areas relative to the reference sub-areas according to the first-order shape function. The calculation formula is as follows:

(3) $$x_{i}^{\prime }=x_{i}+\xi_{1}\left(x_{i},\;y_{i}\right)$$

(4) $$y_{i}^{\prime }=y_{i}+\eta_{1}\left(x_{i},y_{i}\right)$$

where:

(5) $$\xi_{1}\left(x_{i},y_{i}\right)=\mu +\mu_{x}\Delta x+\mu_{y}\Delta y$$

(6) $$\eta_{1}\left(x_{i},y_{i}\right)=\nu +\nu_{x}\Delta x+\nu_{y}\Delta y$$

According to the displacement field obtained, the von Mises equivalent strain field is calculated using von Mises yield criterion [38], the calculation formula as shown in (7):

(7) $$\bar{e}=\frac{\sqrt{2}}{3}\cdot \sqrt{A^{2}+B^{2}+C^{2}+6D}$$

where $A=e_{xx}-e_{yy};B=e_{yy}-e_{zz};A=e_{xx}-e_{zz};D=e_{xy}{}^{2}-e_{xz}{}^{2}+e_{yz}{}^{2}$

(8) $$e_{xx}=\frac{1}{2}\tan^{2}\beta ,\;\;e_{yy}=\frac{1}{2}\tan^{2}\alpha ,\;\;e_{xy}=\frac{1}{2}\left(\tan\alpha +\tan\beta \right),\;e_{zz}=e_{xz}=e_{yz}=0$$

### 3.2. Load–Strain Loop Curve Characteristic Judgement Algorithm

The key algorithm of the proposed method is the load–strain loop curve characteristic judgment algorithm. The following analysis gives us the reason why the load–strain loop curve was selected.

**Figure 10:**
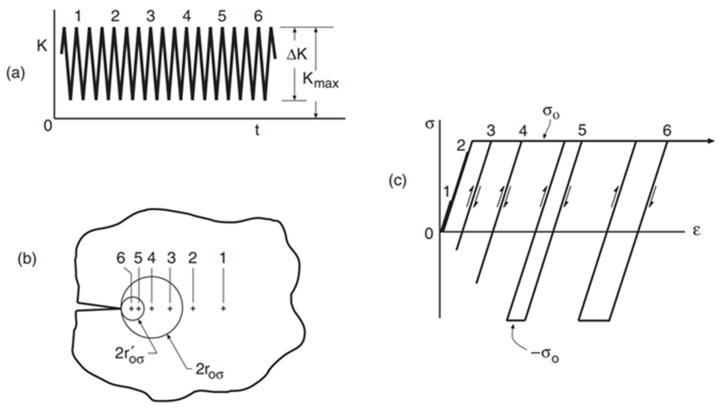
Stress–strain distribution at different distances from the crack tip of ideal material [32]: (**a**) Load waveform; (**b**) measure points layout; (**c**) strain–stress curves.

**Figure 11:**
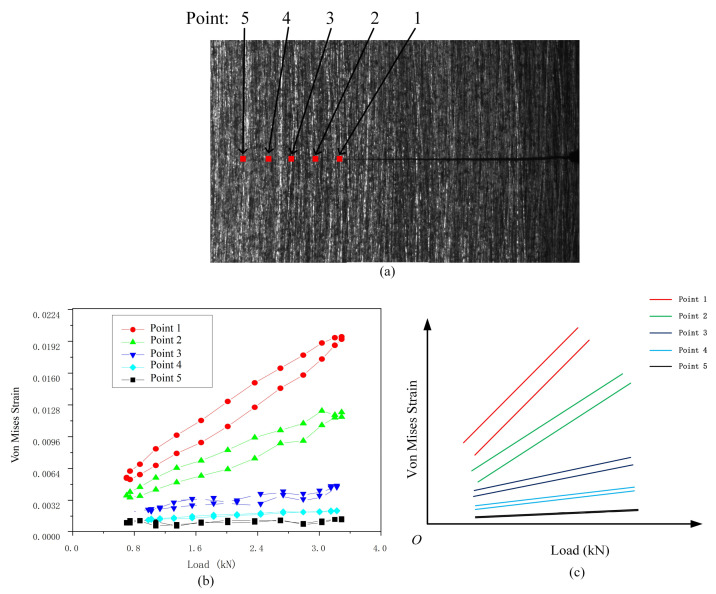
Load–strain distribution at different distances from the crack tip of Q&P 980 steel: (**a**) Measure points layout; (**b**) load–strain curve near the crack tip; (**c**) abstracted load–strain curve.

The stress–strain curves of ideal elastic-plastic materials at different deformation regions are shown in Figure 10 [32]: Points 1 and 2 are located within the elastic region, and the stress–strain curves of the loading and unloading phases appear as coincident straight lines. Points 3 and 4 are located in the monotonous plastic zone, where plastic deformation occurs, but there is no reverse yield. The strain–stress curve of the loading and unloading phases still shows a coincident curve. Points 5 and 6 are located in the CPZ, and there is a reverse plastic zone. The annular stress–strain curve shows a gradual expansion as it approaches the crack tip. Therefore, it is theoretically possible to distinguish different deformation regions by judging the morphology of the stress–strain curves, but it is difficult to obtain stress because a suitable constitutive relation is hard to obtain. However, it is known from [39] that the load–strain curve also has the above characteristics. The load–strain curves at the points near the crack tip region of the CT specimen under a certain crack length obtained in our previous tests are shown in Figure 11, and it is observed that the load–strain curves have the above characteristics; therefore, the load–strain curve can replace the stress–strain curve as the judgment object.

**Figure 12:**
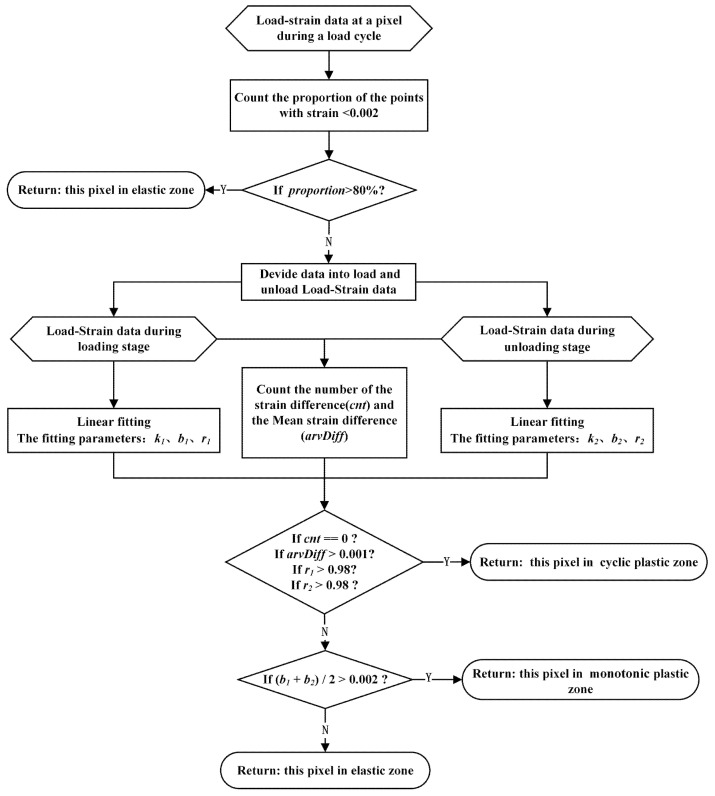
Load–strain loop curve characteristic judging process.

The load–strain curve characteristic judgment algorithm was used to judge the deformation region type according to the different characteristics of the load–strain curves of the pixel points in the crack tip region. There were 26 speckle images in each group of cyclic loads; hence, there were 26 observation points in the load–strain curve of each pixel within the crack tip strain field. The judgment process is shown in Figure 12. First, the yield strain value was taken as 0.002; if the proportion of the observed points in which the strain was less than the yield strain value in a load cycle was greater than 80%, the point was considered to be an elastic zone. For the plastic zone, the observation points were divided into the loading group and the unloading group according to the load value. For each observation point, the unloading strain value of the point was subtracted from the loading strain value; the *cnt* represents the number of differences less than 0, and the *avrDiff* represents and the average difference value. Then, the loading data and the unloading data were fitted by straight-line fitting, respectively, and the slope (*k1, k2*), intercept (*b1, b2*), and correlation between the fitting line and the original data (*r1, r2*) were obtained. If *cnt* > 0, the unloading curve was above the loading curve, and if *arvDiff* > 0.001, the unloading curve was far away from the loading curve. If *r1* > 0.98 and *r2* > 0.98, the linearity of fitting data was good. If the above conditions were satisfied, it was considered that the curve accords with the annular characteristic and was judged to be a CPZ. Otherwise, it was considered that the loading curve and the unloading curve coincided with each other, and the average intercept of the two lines was further calculated. If the intercepts were greater than the yield strain, the point was considered to be a monotone plastic zone, and if it was less than the yield strain value, it was considered to be an elastic zone.

**Figure 13:**
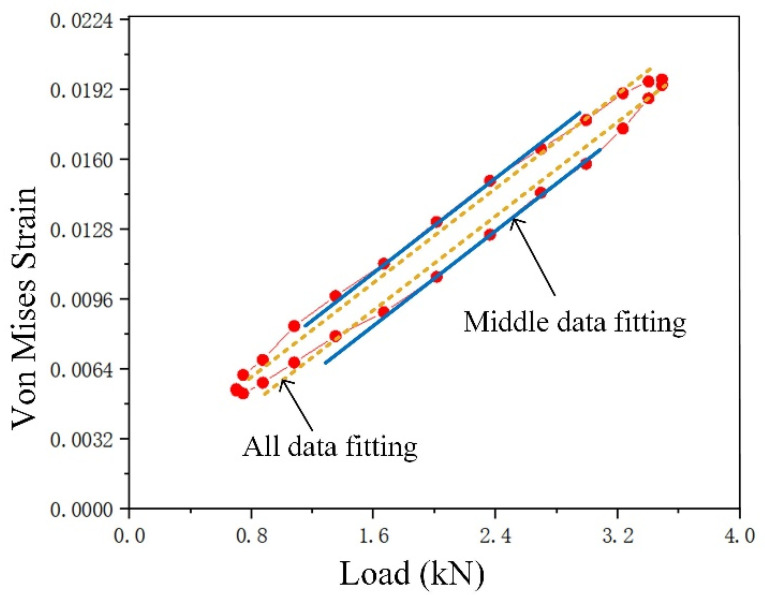
Fitting result of straight-line segment of load–strain loop curve.

The key part of the algorithm is the straight-line fitting algorithm of load–strain curve data, which can simplify the load–strain curve into two straight lines, which represent the loading segment and the unloading segment, and finally judge the characteristics of the load–strain curve type according to the characteristics of the two lines. In this paper, the Random Sample Consensus (RANSAC) straight-line fitting algorithm was adopted, which has the characteristic of automatically selecting the correlation fitting region and can avoid the error influence caused by the direct fitting of the whole segment of data. The results of partial straight-line fitting and whole segment data fitting are shown in Figure 13. It can be seen that the fitting effect of the RANSAC algorithm is better.

## 4. Results and Discussion

### 4.1. Measurement Result of the CPZ near the Fatigue Crack Tip in Q&P980 Steel

**Figure 14:**
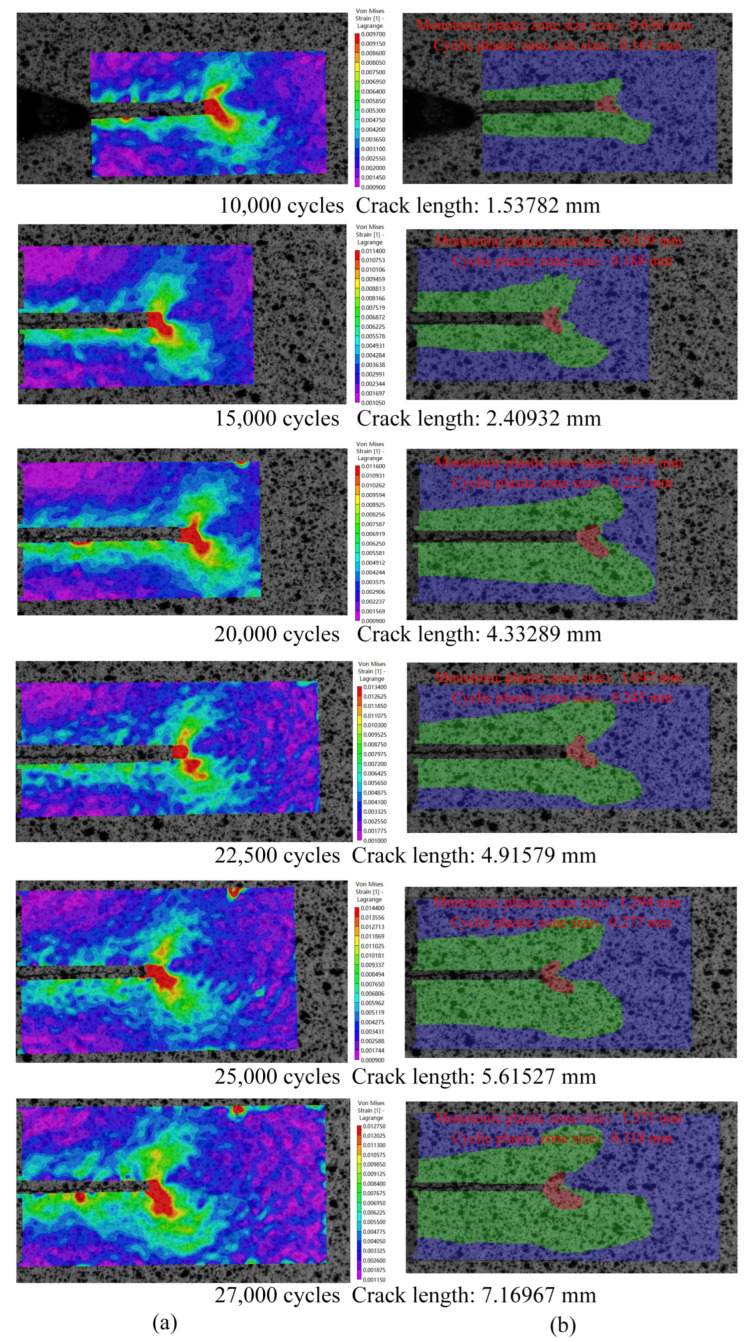
Measurement result: (**a**) Von Mises strain fields near the crack tip under maximum load points; (**b**) cyclic plastic zones near the crack tip under maximum load points of different crack lengths.

**Figure 15:**
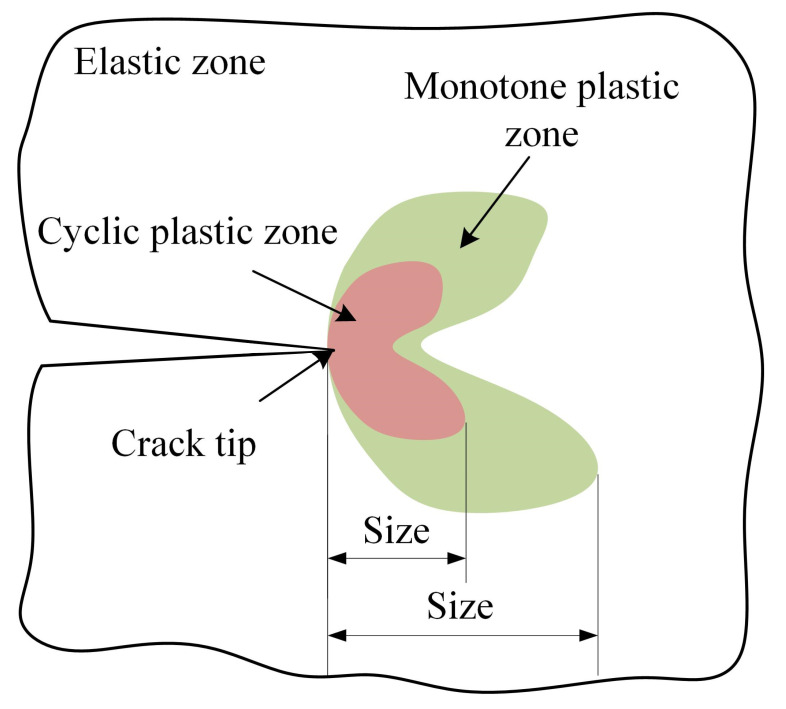
The definition of plastic zone size.

The target images and corresponding reference sub-images were used to perform DIC calculation, and the von Mises strain fields results of data from six groups at the maximum load points are shown in Figure 14a. Based on the load–strain loop curve characteristics judgement algorithm, the results of the CPZ are shown in Figure 14b. The definition of the size of the plastic zone is shown in Figure 15. According to the reference [29], the measurement results of the strain fields are right.

It was found that the shape of the CPZ resembled butterfly wings. This result appears to be similar to that of the existing research [40]. In the process of crack propagation, there was a monotone plastic zone wake along the crack propagation path, and the butterfly wing shape of the CPZ and the monotone plastic zone expanded continuously.

The continuous shrinking of the monotonic plastic zones in the middle part resulted in the elastic zones gradually approaching the CPZ. It is obvious that the growth rate of the CPZ was less than the monotonous plastic zone.

### 4.2. Measurement Results of the CPZ in Q&P980 Steel Verification and Analysis

A comparison of the measured and theoretical results of the CPZ are shown in Table 3, and the theoretical CPZ size was calculated from the computational Formulas (10) and (11) derived from the linear elastic fracture mechanics (LEFM) theory [32]. Formulas (9) and (10) were derived under the condition that the loading state of the specimen was a plane stress state, and the strain in the Z direction was negligible. In addition, it was assumed that the stress intensity factor (*K*) was the main factor affecting the stress, and the final formula calculation result was doubled based on experience. The theoretical values were more than the measured results of the CPZ. This can be considered to be caused by the hardness characteristic of resistance to deformation of the Q&P980 [41]. The results showed the same trend after repeated experiments.

(9) $$2r_{o\sigma }^{\prime }=\frac{1}{\pi }{\left(\frac{\Delta K}{2\sigma_{0}}\right)}^{2}$$

(10) $$2r_{o\sigma }=\frac{1}{\pi }{\left(\frac{K_{max}}{\sigma_{0}}\right)}^{2}$$

where ΔK is the range of stress intensity factor, and $K_{max}$ is the maximum stress intensity factor. Formula (9) is the calculation formula of the CPZ, and (10) is the calculation formula of the monotone plastic zone.

In order to prove that the measured results are consistent with the reality, the difference of surface hardness between the CPZ and monotone plastic zone can be known from reference [20], and the boundary of the CPZ can be determined by looking for the abrupt position of hardness.

**Figure 16:**
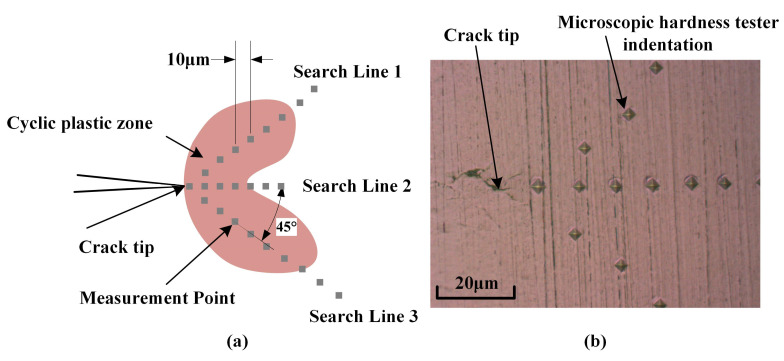
Micro-hardness measurement points position: (**a**) Schematic diagram of hardness measuring points layout; (**b**) the actual indentation of the measuring points.

**Figure 17:**
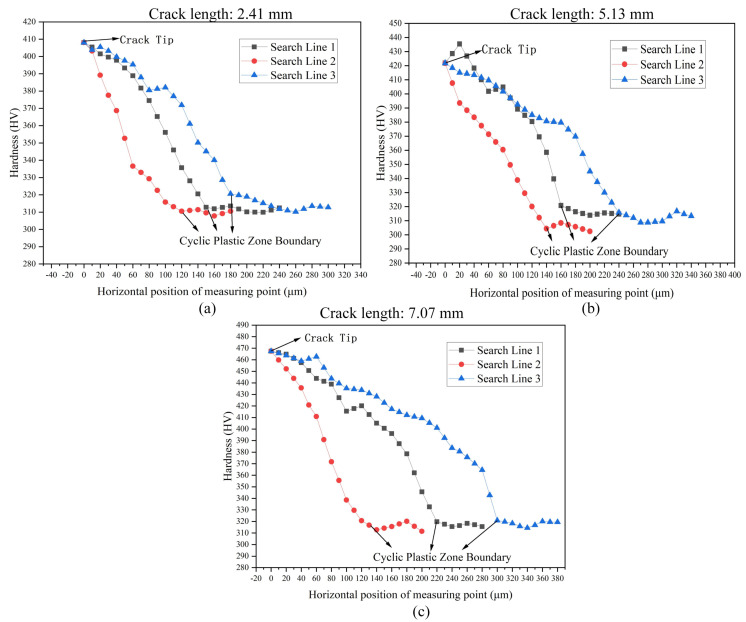
Hardness distribution on three search lines with different crack lengths: (**a**) Crack length: 2.41 mm; (**b**) crack length: 5.13 mm; (**c**) crack length: 7.07 mm.

In addition, three replicate CT specimens were prepared for the FCG test, and the cracks were propagated close to lengths of 2.4 mm, 4.9 mm, and 7.1 mm, respectively. The hardness measuring points were arranged according to Figure 16, and the hardness results are shown in Figure 17. The results show that the hardness distribution from the CPZ to the monotonic plastic zone decreased rapidly to smooth. The overall hardness distribution trend was consistent with the reference [20]. The boundary of the CPZ was determined according to the hardness results, which were compared with the measurement results of the proposed method in this paper. The results are shown in Table 4, and the results of the two measurements are basically similar and reliable. It can be seen that the relative error is basically within 5%, and it is worth noting that this error does not consider the error caused by the micro-hardness test. The hardness method can be considered reliable because the hardness method has been used to study the plastic zone [8,20]. According to the results of the analysis, it can be considered that the method proposed in this paper is reliable.

### 4.3. The Area Evolution Law of CPZ and the Dynamic Response in CPZ in Q&P980 Steel

#### 4.3.1. The Area Evolution Law of CPZ in Q&P980 Steel

**Figure 18:**
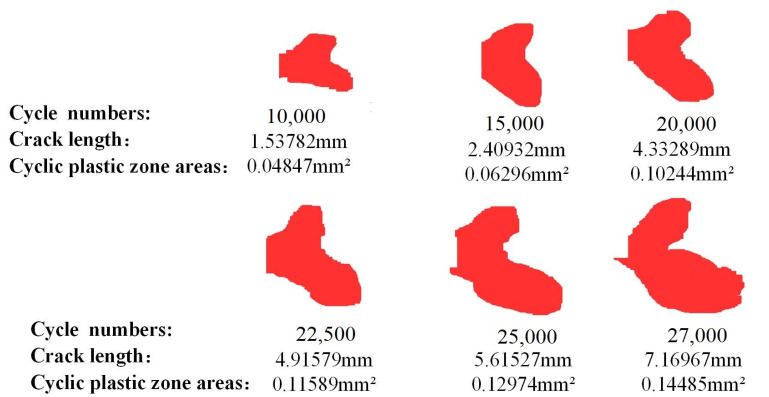
Shape and area of the CPZ under different crack lengths.

**Figure 19:**
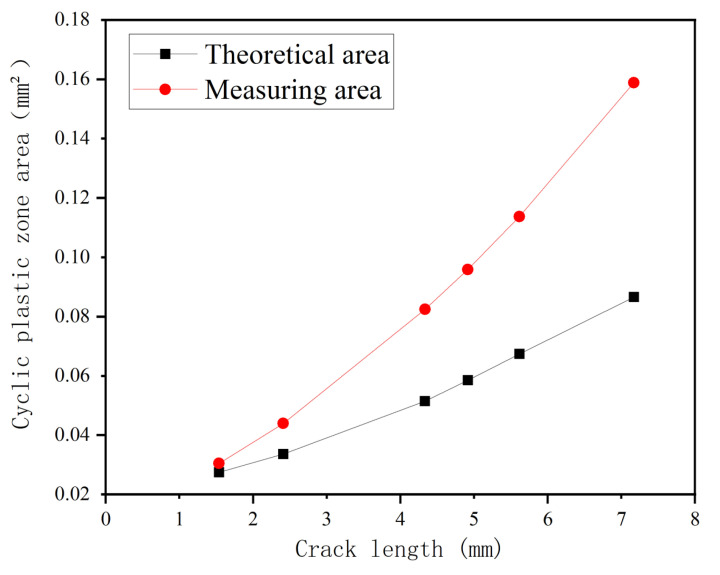
Comparison of theoretical area and measured area of cyclic plastic zone.

In order to analyze the evolution law of the area of the CPZ, Figure 18 shows the area of the CPZ measured at different crack lengths. It is obvious that the area of the CPZ gradually increased. At the same time, theoretical and measured CPZ area curves are plotted as shown in Figure 19. It can be seen that the measured results are always larger than the theoretical value, which is consistent with the analysis in Section 4.2 and can also be attributed to the hardening phenomenon of the material [42,43]. It is also important to note that part of the reason why the measured area is larger than the theoretical area is that the theoretical area is derived from the circular area formula, and the area is covered within the area coverage of the measured CPZ.

#### 4.3.2. Strain Response in CPZ in a Load Cycle at Different Crack Lengths in Q&P980 Steel

**Figure 20:**
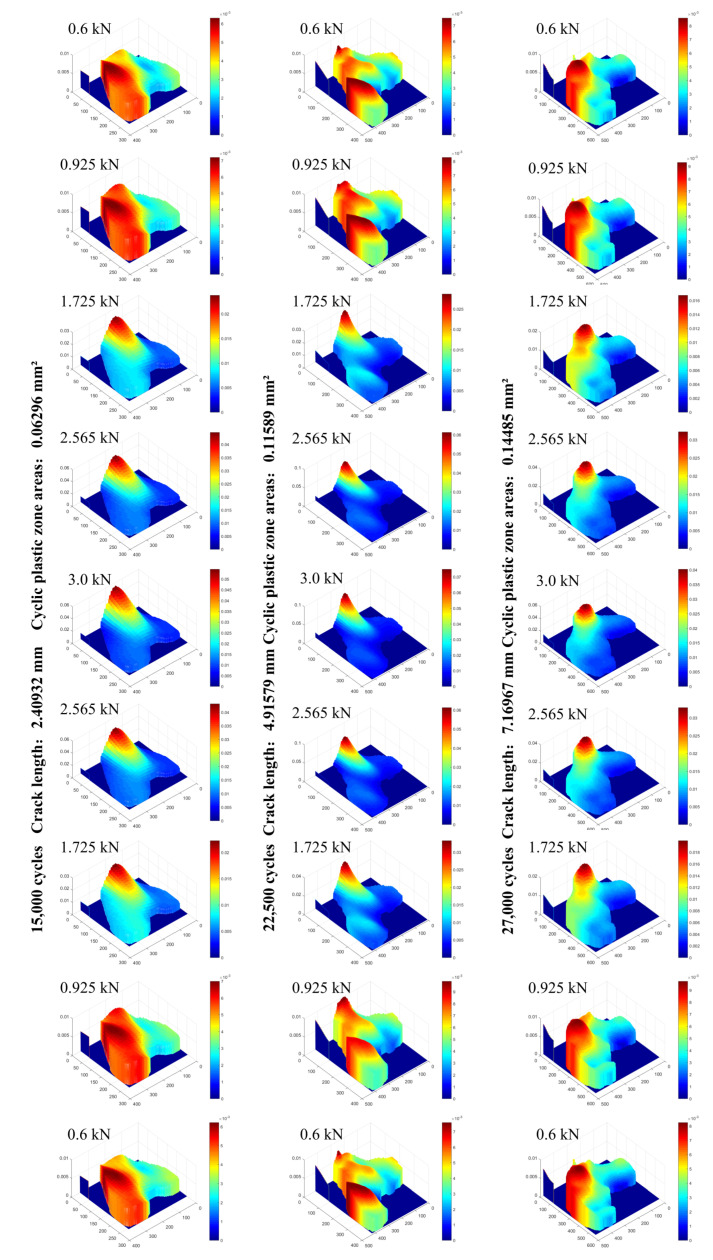
Strain response in the CPZ during a load cycle at different crack lengths.

In order to explore the strain response of the CPZ with different crack lengths during a load cycle, three groups of strain data in the CPZ were extracted to draw the surface diagrams shown in Figure 20. It can be seen that the strain gradient was larger and the strain concentration more pronounced near the maximum load point during a load cycle; the specific performance was the emergence of a peak. It is worth noting that the strains of the loading section and the unloading section were asymmetric. The increasing rate of strain was greater than the decreasing rate of strain in a loading cycle, which indicates that at the same load value, the strain in the loading section was less than that in the unloading section. On the other hand, as the crack propagated, the strain/stress concentration at the crack tip at maximum load became more pronounced [44,45].

#### 4.3.3. Load–Strain Response in CPZ during FCG Process in Q&P980 Steel

**Figure 21:**
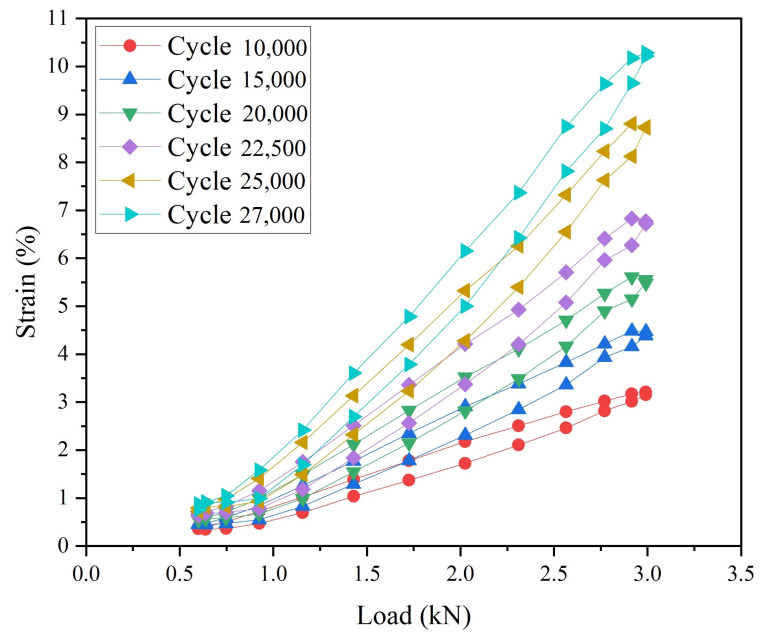
Load–strain response curve at crack tip.

The load–strain curves at the crack tip from the six sets of data are shown in Figure 21. The results show that, as the number of cycles increased (fatigue crack growth), the slope of the load–strain loop became larger, and the area of the load–strain loop became larger. At the same time, the minimum strain of the load–strain loop was basically the same, but the maximum strain gradually increased, which shows that the material gradually yielded, and the strain accumulation gradually increased [46,47].

**Figure 22:**
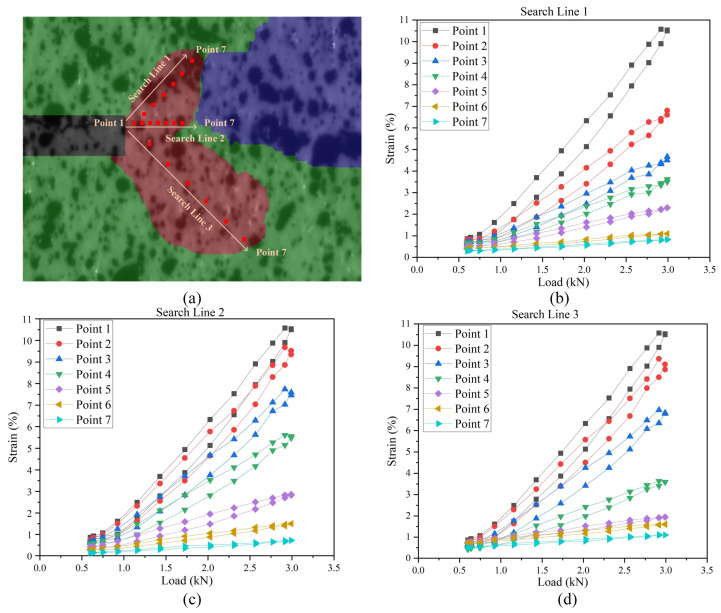
Load–strain curves at the different positions in the CPZ: (**a**) Arrangement of measuring points; (**b**) load–strain curves of search line 1; (**c**) load–strain curves of search line 2; (**d**) load–strain curves of search line 3.

In order to fully observe the response of the load–strain curves at different points near the crack tip, the analysis points were arranged according to Figure 22a, and the results of the load–strain loop are shown in Figure 22b–d. The results show that the load–strain loop at the position closer to the crack tip had a larger slope, more maximum strain value and larger area of load–strain loop. It was also found that the decrease in the maximum strain value of the load–strain loop was not uniform, which can be considered to be caused by the complex strain response of the QP980 material.

In addition, it should be noted that, because the load–strain loop curve was used in this study, the measurement results are directly affected by the shape of the load–strain loop curve. The load–strain curve of some materials during deformation is not smooth but shows a serrated wave, such as Al-Mg of Al-Cu aluminum alloys [48,49]. For such materials, it is necessary to optimize the load–strain loop curve judgment algorithm to achieve better measurement.

## 5. Conclusions

In this paper, some further study of our previous research [29] has been presented, which is a new method for in situ on-line measurement of CPZ. This method is not only convenient to describe the CPZ but can also overcome the limitation of the microscopic field of view on the measurement of the CPZ and dynamically track the crack tip to measure the CPZ. The methodology has been successfully applied on Q&P980 steel subjected to cycle load. The main findings can be summarized as follows:

(1) Comparing with the result of the micro-hardness experiment, the results of the CPZ measurements are reliable, and the measurement method proposed in this paper is available.
(2) It was observed that the CPZ near the crack tip showed a shape of butterfly wings, and the phenomenon of strain concentration was obvious near the crack tip in the CPZ. However, it is noted that the area of CPZ was greater than the theoretical value. This can be attributed to the hardening of the material.
(3) The results and the analysis indicate that, with the crack propagation, the size of the CPZ, the degree of the strain concentration near the crack tip, and the area difference between theoretical and actual CPZ all increased. Moreover, within a load cycle, the strain concentration was strongest at the maximum load.
(4) The results have also shown that the area of the load–strain loop at the crack tip at the maximum load gradually increased with the number of cycles. The closer to the crack tip, the larger the area of the load–strain loop.

The novelty of the current methodology lies in the combination of an image stitching and matching algorithm with the load–strain curve feature judgement algorithm and the micro-DIC algorithm. We believe this work contributes to filling the gap of in situ dynamic measurement of the CPZ at the microscopic scale.

## Figures and Tables

**Table 1:** Mechanical properties of Q&P980.

(Young’s Modulus) E/GPa	(Yield Strength) σ/MPa	(Elongation) δ /%	(Strain Hardening Index) n
197.20	776.48	21.33	0.17

**Table 2:** Camera parameters.

Parameters	Microscopic Camera	Macroscopic Camera
Focal length (mm)	105	35
Working distance (mm)	115	232
Field-of-view size (mm × mm)	4 × 2.11	40 × 30
Spatial resolution (μm/pixel)	0.97	19.47

**Table 3:** Measurement results and theoretical results of CPZ in Q&P980 steel.

Cycle Numbers	Crack Length/mm	Size of CPZ/mm		
		Theoretical Values	Measured Values	Errors/%
10,000	1.53782	0.187	0.161	13.904
15,000	2.40932	0.207	0.188	9.179
20,000	4.33289	0.256	0.225	12.109
22,500	4.91579	0.273	0.245	10.256
25,000	5.61527	0.292	0.277	5.137
27,000	7.16967	0.332	0.318	4.217

**Table 4:** Comparison of two different methods of CPZ measurement.

Crack Length /mm	The Horizontal Distance from the Boundary of the CPZ to the Crack Tip/μm								
	Search Line 1			Search Line 2			Search Line 3		
	Proposed Method	Hardness Method	Error %	Proposed Method	Hardness Method	Error /%	Proposed Method	Hardness Method	Error /%
2.41	156	150	4.00	124	120	3.23	189	180	5.00
4.93	168	160	1.25	145	140	3.57	250	240	4.00
7.07	231	220	5.00	133	130	2.31	308	300	3.00

## Data Availability

Not applicable.
